# Facilitators and barriers to the sustainability of universally accessible parental training interventions: a systematic qualitative review using normalization process theory

**DOI:** 10.3389/fped.2026.1802287

**Published:** 2026-08-27

**Authors:** Mette Louise Petersen, Hedvig Ramnefjell Dolva, Sarah Strøyer de Voss, Ida Scheel Rasmussen, Maiken Pontoppidan, Ruth Kirk Ertmann, Philip Wilson, Gritt Overbeck

**Affiliations:** 1 Research Unit for General Practice & Section of General Practice, Department of Public Health, University of Copenhagen, Copenhagen, Denmark; 2 VIVE—The Danish Center for Social Science Research, København K, Denmark

**Keywords:** child health, implementation science, mental health, parent-child relations, primary health care

## Abstract

**Background:**

Universal parenting programmes aim to strengthen parent-child relationships and support parental well-being during the transition to parenthood. While these programmes can improve parental confidence, self-efficacy, and child social-emotional development, achieving sustained delivery in routine practice remains a major challenge. Understanding the factors that facilitate or hinder their implementation is essential for optimizing their public health impact. This review synthesizes qualitative evidence on barriers and facilitators to the implementation and sustainability of universally accessible parental training interventions.

**Methods:**

We conducted a systematic qualitative review following PRISMA guidelines. Ten databases (PubMed, PsycInfo, SocIndex, CINAHL, Embase, Medline, Sociological Abstracts, ERIC, Scopus, and Web of Science) were searched in May 2024, and updated in July 2025, for peer-reviewed qualitative studies published after 2003 in English. Studies were eligible if they evaluated universal parenting interventions in high-income countries and explored aspects of implementation or sustainability. Interventions targeting specific clinical populations were excluded. Data extraction was conducted by multiple authors, and reporting transparency was assessed using the Consolidated Criteria for Reporting Qualitative Research (COREQ). Findings were synthesized using Normalization Process Theory (NPT) to identify how interventions become embedded in practice.

**Results:**

Eight studies from six countries were included, covering interventions delivered in primary care, community health, and web-based settings. Across NPT constructs, key facilitators included strong stakeholder engagement, peer and managerial support among participants, and flexible delivery models that fit local contexts. Recurrent barriers were limited time and resources, insufficient training and weak managerial or policy support. Parent engagement was sometimes constrained by perceptions of limited programme relevance or logistical obstacles, highlighting the importance of clear communication and iterative adaptation to local needs.

**Conclusions:**

Universal parenting interventions can contribute to improved parental and child outcomes when effectively integrated into routine practice. Sustainable implementation depends on early co-design with stakeholders, alignment with existing health services, and sustained managerial and policy support. Embedding feedback loops and mechanisms for adaptation can strengthen engagement and ensure that interventions remain feasible and relevant over time. Future research should focus on strategies to enhance reach and to assess long-term population-level effects on child well-being.

**Systematic Review Registration:**

https://www.crd.york.ac.uk/PROSPERO/view/CRD42024559474, identifier CRD42024559474

## Introduction

In many countries, increasing rates of mental health problems are a major concern, and in high income countries, an estimated one in eight children aged eighteen or younger have been diagnosed with a mental disorder (1). Research shows a protective effect of positive parent-infant interaction against childhood psychological problems (2–6), and that parental mental well-being is a major determinant of children's healthy social and emotional development (7, 8). The early parent-infant relationship plays a crucial role in nurturing the child's emotional well-being and supporting healthy development (9–11). Healthy early relationships are also the key to healthy brain development and long-term well-being (12). The transition to parenthood can be experienced as overwhelming and stressful. Interventions focusing on strengthening parenting knowledge and skills through parental training have been shown to help parents adjust to their new life circumstances and to improve parent-infant relationships (13, 14). Implementing interventions that support parents in early parenthood and enhance both parental mental health and the parent-child relationship are therefore important strategies to promote child development and represent valuable public health interventions (15, 16).

Much research supports the success of targeted parenting programmes for families experiencing challenges (16, 17). Universal parenting programmes are public health interventions offered to all parents regardless of risk status. In universally offered programmes even small average effects can yield substantial population-level benefit—the prevention paradox. The effectiveness of universal parenting programmes is debated, as some studies find no evidence for positive effects of universal parenting interventions (18), whereas other indicates increased positive parenting behaviours, parental confidence, and parental self-efficacy, while simultaneously reducing harsh disciplinary practices, punitive behaviours, and parental stress (19, 20). In addition, some universal parenting interventions have demonstrated potential benefits in improving children's emotional regulation and social skills, as well as reducing their problematic behaviours (19, 20). Universal parenting programmes are perceived as less stigmatizing, given their inclusive approach to family support and have the capability to engage families who may otherwise remain undetected (19–21). The impact of parenting programmes is contingent upon the quality of their implementation. Little is known, however, about how parenting programmes are implemented in real-world settings (22), which underscores the importance of understanding the barriers and facilitators to implementation. Implementation phases can be viewed through different frameworks and theories (23). In this review, we understand sustainability as the continued delivery of an intervention as part of routine practice after the initial implementation period, encompassing both maintenance of core components and integration into existing workflows. Qualitative systematic reviews can be used to collect and summarize existing knowledge of implementation processes and challenges (24). The aim of this qualitative systematic review is to examine barriers and facilitators to implementation and sustainability of universally accessible parental training interventions for parents of children aged 0–6 years.

## Methods

The protocol for this review was registered in PROSPERO (ID: CRD42024559474).

### Search strategy and sources

A systematic and comprehensive search was conducted 12th May 2024 and updated 30th July 2025 in the following 10 databases: PubMed, PsycInfo, SocIndex, CINAHL, Embase, Medline, Sociological abstract, ERIC, Scopus and Web of Science. The search strategy (see full search string in Supplementary File 1) was constructed using a combination of terms across three distinct conceptual categories, linked by the Boolean operators OR (within categories) and AND (between categories). The categories comprised: 1) Population (e.g., parent), 2) Phenomena of Interest (e.g., interaction) and 3) Context (e.g., primary care) (25). Terms were applied to searches in both titles and abstracts, and, where applicable, supplemented by database-specific thesauruses to enhance precision. When applicable, filters for children in the database were used.

### Study selection

Three authors (MLP, HRD, GO) performed the study selection following PRISMA guidelines. First, titles and abstracts were screened by two authors and disagreements were resolved jointly. Selected articles were screened as full texts and data were extracted accordingly.

### Inclusion and exclusion criteria

In addition to exploring elements of implementation or sustainability for interventions promoting parent-child relationships, search criteria included interventions with potential to be universally accessible (offered to all parents in a defined population, regardless of economic and health risk status). Interventions accessible within larger demographic groups were included. Furthermore, the following exclusion criteria were applied: middle- and low-income countries, interventions targeting parents or children with specific and/or severe illness, cognitive disabilities etc. (e.g., postpartum depression, cancer), interventions targeting children, families considered at-risk, and quantitative studies. The studies had to be peer-reviewed, published within the last 20 years (2004–2025) and available in English. See Table 1 for inclusion and exclusion criteria.

**Table 1:** Inclusion- and exclusion criteria.

Inclusion criteria	Exclusion criteria
Population	
Universally accessible programmes including interventions targeting demographic groups.	Middle- and low-income countries Interventions targeting parents or children with specific and/or severe illness, cognitive disabilities etc. (e.g., postpartum depression, cancer) or families considered at-risk in other ways Interventions targeting children
Design	
Studies exploring elements of implementation or sustainability for interventions promoting parent-child relationships Qualitative studies and mixed-methods studies	Other study designs including quantitative studies
Publication type	
Peer-reviewed studies published after 2003, available in English.	

### Quality assessment

Two authors (MLP and HRD) applied the consolidated criteria for reporting qualitative research checklist (COREQ) to perform an assessment of the quality of transparency in the included studies (26). Uncertainties arising during this process were discussed and resolved in collaboration with a third author (GO).

### Data extraction

For all included studies, we extracted data on study characteristics including country, number of respondents and data collection methods, characteristics of the parental training programme and findings about facilitators and barriers to implementation. In mixed-methods studies, we only extracted the available qualitative data. The qualitative synthesis was based on the findings (results) sections of the included articles—author-reported results and participant quotations—while descriptive characteristics were extracted separately for Table 2. This included country, number of respondents and data collection methods, and characteristics of the parental training programme.

**Table 2:** Overview of the characteristics of the eight included studies.

Author, country	Aim	Name and details of intervention study	Accessibility, target group	Study setting (health professionals)	Respondents and data collection method
Baldwin et al. (29), UK	The study aimed to explore health visitors' use of the programme Promotional Guide with fathers, their assessment of father's mental health and facilitators and barriers when using the programme in practice.	Promotional Guide (PG) - Antenatal (11 topics) and postnatal 6–8 weeks (10 topics) - Designed to support both parents - Conversation with health professionals - For each topic, the guide outlines key facts, risk factors and factors promoting resilience, followed by topic prompts and actions.	Universal, mothers and fathers	Through the national Healthy Child Programme (health visitors)	18 health visitors from four London districts served by two National Health Service organizations were included through 11 interviews and 7 observations using PG in practice
Callejas et al. (30), Canary Isles, Spain	The study has a qualitative and a quantitative aim, whereas the qualitative aim is to explore facilitators' perspectives of the initial implementation of the hybrid version of the GH&W programme.	Gaining health and wellbeing from birth to three (GH&W) - The focus is on the development of stimulating daily routines and healthy parent-child activities - 3 levels of intervention in the programme 1) L1: online version of GH&W 2) L2: L1 + four face-to-face group workshops 3) L3: L2 + individual support at regular paediatric check-ups.	Universal and selective, parents	Public primary healthcare centres (nurses, paediatricians and social workers)	50 healthcare professionals from 17 public primary healthcare centres completed a survey* and of those 31 participated in 6 focus group (L2 = 12 healthcare professionals, L3 = 19 healthcare professionals)
Connor et al. (31), Baltimore	The aim was to assess stakeholder perspectives on facilitators and barriers, as a measure of the feasibility of implementing CP in FQHCs in Baltimore.	Centering Parenting (CP) - Group with 6–8 dyads (mother and infant) - 8 sessions - Parents learn care skills, participate in facilitated discussions, and develop a support network with other group members.	Low-income population, mother and infant	Federally qualified health centres (FQHC) (two clinicians at each CP, e.g., paediatrician, family physician, nurse practitioner, and e.g., social worker, lactation consultant)	Interviews with 26 mothers and 16 clinicians, staff and administrators
Hickey et al. (32), Ireland	The aim of the study is to provide an overview of the development and setting up of the PIN programme including identification of the facilitating and inhibitive factors influencing both its roll-out and the partnership working in the initial phases of implementation	Parent and Infant Programme (PIN) - A group-based early parenting intervention - The aim is to provide education and supports to improve parent competency and well-being, strengthen parent-child relationships and enhance child developmental outcomes - Components: Two Incredible Years parenting programme workshops and classes aimed at educating and supporting new mothers	Universal, parents	Primary health care and community-based services	Documentary review* and interviews with 19 key informants involved in programme development and implementation planning and including community-based service managers (*n* = 4), Public Health Nurses and Nurse Managers (*n* = 6), family support workers and community-based practitioners (*n* = 9)
Hickey et al. (33), Ireland	The aim of the study was to explore the successes and challenges of implementing the PIN programme-in real-world, community-based settings.	Parent and Infant Programme (PIN) The intervention is the same as Hickey et al. (32)	Universal, parents	Primary health care and community-based services	Documentary analysis*, Interviews (Mothers (*n* = 22), Community-based service managers (*n* = 4), Public health nurses and nurse managers (*n* = 6), family support workers and community-based practitioners/volunteers (*n* = 12) Focus group with parents (*n* = 1) and with programme implementers (*n* = 6) including (Mothers (*n* = 5), fathers (*n* = 1), programme facilitators and implementers (*n* = 18)
Patterson et al. (34), UK	The aim is to report the usefulness of the programme to the parents, aspects they found helpful and why, and the extent to which they had observed changes in their own and their children's mental health and behaviour as a result of the programme.	Webster-Stratton Parents and Children Series parenting programme courses - Video-based course, 10 weeks, 2 h each week - Four modules of step-by-step guidelines for playing with children and helping them learn, using praise and rewards, setting limits and handling misbehaviour. - Parenting groups of 10 parents run by two professionals, at least one health visitor	Parents with children who scored in the worst half of the distribution in the Eyberg Child Behaviour Inventory. Children with learning difficulties or who were receiving treatment for behaviour problems were excluded.	General practice	Questionnaire, tape recordings of supervision meetings and interviews with parents (*n* = 26)
Saxild et al. (35), Denmark	The aim of the study was to explore the attitudes and experience of general practice clinicians who directed their patients to the Resilience Programme web-portal.	Resilience Programme (FamilieTrivsel) - Web-based psychoeducational programme - Flexible modules offering information about resilience, mentalization, stress-management and cognitive training	Universal, mothers	Web-based intervention, informed at primary preventive antenatal consultation in general practice	Interviews with clinicians; general practitioners (*n* = 11), practice midwives (*n* = 3), practice nurse (*n* = 1)
Westerlund et al. (36), Sweden	The overall aim was to investigate the involved actors' views on factors likely to affect the initial implementation of ICDP and strategies used to manage them.	International Child Development Programme (ICDP) - The ICDP is non-instructive and aims to guide caregivers' interactions with children	Universal, mothers and fathers	A county council with 40 health centres providing tax-funded healthcare services	Questionnaires* Interviews (*n* = 21) with preschool teachers (*n* = 3), child health nurses (*n* = 2), midwife (*n* = 1), social worker (*n* = 1), HC managers (*n* = 6), CHC managers (*n* = 2), CHC psychologists (*n* = 4), Salut facilitators (*n* = 4)

* Data not used in this review due to lack of availability or data being quantitative.

### Data analysis

We used a directed (deductive) qualitative content analysis with the Normalization Process Theory (NPT) constructs as the coding frame (27, 28). NPT provides a robust lens for understanding how complex interventions become embedded and sustained in routine practice by examining four key constructs: **coherence** (how stakeholders understand and make sense of the intervention, e.g., staff understanding the programme's purpose), **cognitive participation** (how stakeholders engage with the intervention, e.g., staff and parents choosing to engage), **collective action** (how the intervention is enacted in practice, e.g., the practical work of delivering it), and **reflexive monitoring** (how the intervention is appraised and adapted over time) (27).

The initial coding of data was performed through a directed (deductive) qualitative content analysis (28) with the four NPT constructs as *a priori* categories. One author (MLP) conducted the coding using NVivo 14, and the process was iteratively refined through discussions with a second author (GO) to ensure consistency and reflexivity in the analytical approach. Data segments that did not clearly align with the four core constructs were discussed collaboratively and re-interpreted within the NPT framework. Coded segments were additionally classified as facilitators or barriers. This approach enabled a theory-informed synthesis of the barriers and facilitators to implementation and sustainability across diverse intervention contexts.

## Results

### Study characteristics

**Figure 1:**
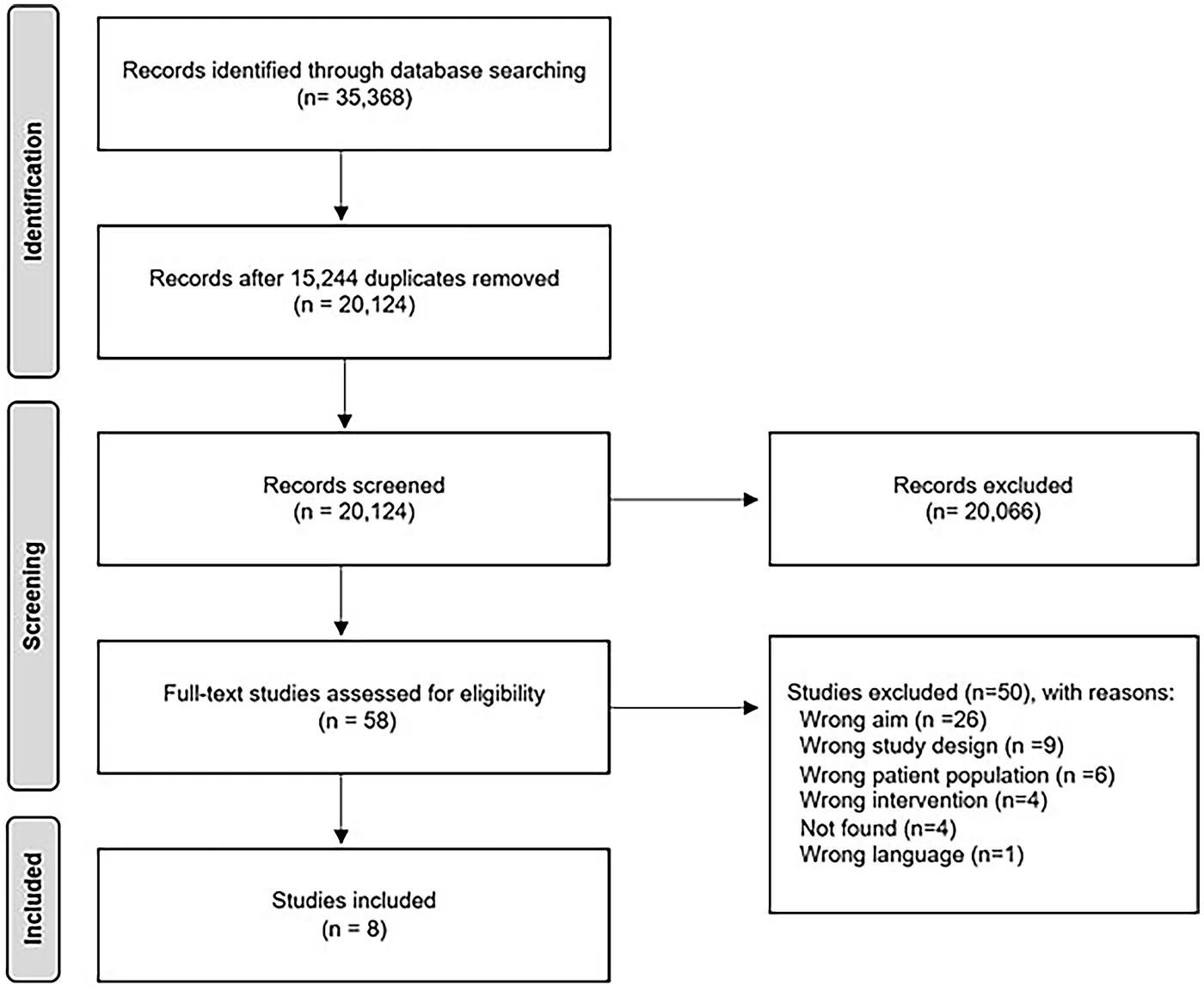
The PRISMA flow diagram for the qualitative systematic review.

As shown in the PRISMA flow diagram below (Figure 1), 20,124 studies were identified after the removal of duplicates. 20,066 studies were excluded after title/abstract screening and 50 studies were excluded after full-text screening, primary due to wrong aim, wrong study design or wrong patient population. Eight studies were selected for inclusion in this qualitative systematic review. The small number of eligible studies itself reflects the scarcity of qualitative implementation research on genuinely universal programmes: most records were excluded for reporting only quantitative outcomes or targeting at-risk populations. The included studies were conducted across the United Kingdom, Ireland, Sweden, Denmark, Spain, and the United States and published between 2005 and 2024. The interventions included conversational tools, group-based parenting programmes, hybrid digital-in-person models and fully web-based psychoeducational programmes. Across studies, the programmes targeted parents of infants and young children and were typically delivered by health visitors, public health nurses, general practice clinicians or community practitioners. Data collection methods were predominantly qualitative, often based on interviews or focus groups with health professionals and parents. Several studies supplemented qualitative data with surveys or documentary analyses. Respondents included a wide range of stakeholders involved in programme delivery or participation, such as health visitors, nurses, midwives, general practitioners, programme facilitators and parents themselves. The characteristics of the eight included studies are shown in Table 2.

### Quality assessment

The COREQ scores ranged from 4 to 19, with a mean of 15.5 and a median of 17. Although the COREQ scores indicate variability in reporting quality, all studies were included in the synthesis.

### Results of the qualitative synthesis

The results are presented according to the four constructs of NPT: Coherence, Cognitive Participation, Collective Action and Reflexive Monitoring. The construct Collective Action is most comprehensively covered and is further described with the four sub-components which are part of the original NPT formulation of Collective Action (27): *Interactional Workability* (the everyday work of using the intervention in interactions between professionals and parents), *Relational Integration* (the work of building and sustaining the knowledge and trust that hold the new practice together), *Skill Set Workability* (provider skills and competence) and *Contextual Integration* (organisational resources and support) (27). Table 3 provides an overview of the most covered facilitators and barriers in each NPT-construct.

**Table 3:** The most covered facilitators and barriers in each NPT component.

NPT construct	Facilitator	Barrier
Coherence	Sense-making among health professionals (30, 33, 35, 36) Better communication with parents (29, 30, 33)	Lack of initial information for health professionals (30, 36)
Cognitive participation	Stakeholder buy-in (29–36) Group-based delivery and supportive group environment (31, 33, 34)	Experienced lack of time among health professionals (29, 33, 35) Recruitment barriers (29, 30, 33)
Collective action	Improved communication through specific tools (29, 32, 34, 35) Adequate professional skills (29, 35) Inter-organizational collaboration and support (29, 30, 32, 33, 35, 36)	Insufficient training and resources (29, 30, 33, 36) Time constraints (29, 30, 33, 35, 36)
Reflexive monitoring	Proactive implementation planning (32, 33)	No systematic follow-up or evaluation (29, 34, 36)

#### Coherence

The studies presented few findings about how professionals and parents initially made sense of the interventions. Some studies explicitly reported acceptability and sense-making of the interventions among health professionals (30, 33, 35, 36), e.g., in Saxild et al. where a health professional stated: “*I believe that there exists a general lack of focus on mental well-being in families with children—not just in general practice, throughout the healthcare system”* (p. 3) (35). Some health professionals requested sufficient initial information about the programmes (30, 36). For example, some practitioners only received information about the programme on the first day of training, stating: “*We really do not know what is going to happen next because we felt from the beginning that we had not received any information”* (p. 134) (36)*.* Some studies reported that health professionals experienced better communication with the parents after introducing the intervention (29, 30, 33). For instance, in the study of Callejas et al. health professionals stated how the intervention had positively affected their professional development, e.g., through improvements in the collaboration with the parents and more awareness of the parents' needs (30).

#### Cognitive participation

Securing stakeholder buy-in was identified as essential in multiple studies (29–36). The delivery mode (e.g., group-based formats) was often highlighted as a facilitator or barrier to engagement by both parents and health professionals (29–35). Most health professionals found the group-based format beneficial and parents expressed a sense of being acknowledged and supported by peers, feeling less isolated in their role as parents, learning from others in similar situations, or obtaining answers to questions one might not feel comfortable asking oneself, but were raised by others (33, 34). Some mothers were enthusiastic about the diversity of the group (31), while discomfort with group settings and stigma around parenting programmes limited engagement for others (33). The health professionals' ability to establish a warm, non-judgmental and supportive group environment improved parents' engagement and satisfaction (33, 34). Hickey et al. reported some minor differences between health professionals and parents; where health professionals found the programme relevant for all parents, some experienced parents felt the content was less relevant (33) and Saxild et al. found that the programme appealed more to first-time, resourceful mothers than to broader target groups (35). Callejas et al. reported low enrolment rates and low commitment to sessions (30), while Baldwin et al. reported not having a clear system to invite fathers specifically (29) as potential recruitment barriers. Setting influenced experience: a home setting was described as ideal for privacy and more relaxed parents according to the health professionals (29), while transport and lack of childcare support limited attendance (33) and family practice was seen as a supportive setting for conversations (35).

Allocated time and managerial support was crucial for professionals' willingness to commit to the programme (29, 32, 33, 35, 36). Perceived lack of time hindered engagement in delivering the programme (29, 33, 35), and when preparatory time was provided, it reduced stress and enhanced commitment (33). This is stated in Saxild et al. as: “*Clinicians were initially enthusiastic about introducing the website. However, the level of motivation started to diminish in part because introducing the interventions content was time-consuming”* (p. 3) (35). Ongoing support improved stakeholder's confidence and helped address early fears or challenges and the importance of system-level buy-in for effective implementation was stated in Hickey et al.: “*If you don't have that buy-in from the top, that's what kind of hinders it”* (33).

#### Collective action

##### Interactional workability

Improved communication, supported by specific elements of the interventions, supported implementation (29, 32, 34, 35). The inclusion of fathers was positively noted and the use of topic cards helped guide conversations and facilitate involvement, which were perceived as motivating and supportive for good dialogue (29). Signposting to a website supporting parental mentalization skills might be a helpful conversation starter in general practice, however, Saxild et al. emphasized the importance of sensitive communication when addressing mental health to avoid implying parental inadequacy: “(…) *it became apparent that the clinicians' approach and phrasing was crucial to the outcome of such conversations*” (p. 4) (35). It was also explained that health professionals modified their language based on their perception of the resourcefulness of mothers (35).

##### Relational integration

Open, nonjudgmental dialogue and focus on positive group dynamics promoted relevance, trust and peer support, helping parents realize that others faced similar challenges and learning from each other (33–35) and engaging quiet parents required specific facilitation skills (34). Difficulties in gaining access to fathers and lack of continuity in provider contact was a barrier to implementation (29).

##### Skill set workability

Sufficient training and ongoing support were identified as important for strengthening skill development, and proper training before and during implementation was highly valued (29, 30, 32, 33, 35, 36). Saxild et al. found the intervention feasible and that most professionals could integrate the intervention into routine care, implying they had the relevant skills (35). Baldwin et al. (29) highlighted reliance on clinical judgment for deciding whether to include both parents when discussing certain themes. In several studies, however, health professionals called for additional or more in-depth training (29, 30, 33, 36), e.g., due to them not feeling adequately prepared for the introduced programme as in Westerlund et al., where health professionals expressed ambiguity transferring their new knowledge to their work practice without support (36). Hickey et al. identified insufficient practice as a group facilitators as a barrier, while practical experience of running the groups was helpful to promoting adoption (33).

##### Contextual integration

Most studies described the organisational work needed to fit the intervention into existing routines, including managerial support, time allocation and recognition of workload (29, 30, 32, 33, 35, 36). Resource constraints, costs and policy conflicts made prioritization difficult (33, 36). However, some strategies were effective, such as involving management early (32), inter-organisational collaboration (33), considering how the intervention aligns with the existing practice before deciding to implement the intervention (36), ensuring leadership support (29, 30, 33) and peer support from colleagues not directly involved in the intervention (33). Time constraints emerged as a consistent barrier affecting content depth, participation, and balance with other responsibilities (29, 30, 33, 35, 36). Baldwin et al. illustrated this tension: “*.We understand the values of the promotional guide, (..), but we also have to come back to the office and tick boxes, (..), and it is that balance which is tricky*” (p. 105) (29).

#### Reflexive monitoring

Satisfaction and concerns about the interventions were described across the included studies (29, 30, 32–36). In Patterson et al. parents expressed a need for more support or follow-up sessions after the course ended (34) and in Callejas et al. professionals appraised the programme as valuable, but expressed concern about sustainability as many felt the intervention would be more feasible if activities were embedded in existing schedules (30). Similarly, staff in the PIN studies described how involving key decision-makers, gathering regular feedback from both professionals and parents, and proactive implementation planning supported feasibility and long-term integration (32, 33). In Saxild et al. health professionals appraised the fit and value of the intervention, however they questioned whether the early antenatal visit was the optimal time to introduce the website (35). Some health professionals voiced frustration about the lack of systematic follow-up and evaluation (29, 36). As one participant in Westerlund et al. noted: “*If you never evaluate, you can never know. How can someone put so much money, time, and effort into something, an education or programme or whatever, and not make sure they get what they expect out of it*?” (p. 134) (36).

## Discussion

This review identified a set of interrelated factors influencing the successful implementation and sustainability of universal parenting interventions. Applying Normalization Process Theory (NPT) highlighted that stakeholder engagement, peer and managerial support, and flexible delivery models consistently facilitated embedding interventions in practice. Conversely, structural barriers—such as insufficient time, inadequate training, and weak managerial or policy support—recurrently undermined sustainability. These findings closely mirror insights from broader implementation reviews, including Jürgensen et al., who emphasise the central role of facilitator preparedness, organisational support, and clarity of roles in shaping implementation quality (37). Similarly, Pinto et al. demonstrate that real-world implementation of parenting programmes is often underreported, particularly regarding sustainability, feasibility, and adoption—an issue also apparent in our included studies, where descriptions of implementation processes varied widely (22). This gap underscores the importance of strengthening reflexive monitoring processes and establishing systematic feedback loops to support ongoing adaptation without compromising programme integrity, a point also highlighted in foundational implementation literature (38, 39).

Although universal intervention effect sizes are typically smaller than in targeted interventions—likely due to the lower baseline risk in the general population—universal approaches can offer distinct public health benefits (the prevention paradox) (19, 20, 40). Notably, they have the capacity to engage families who may otherwise remain undetected and are less likely to seek assistance independently (19, 20). In addition, universal preventive programmes are also perceived as less stigmatizing, given their inclusive and non-selective approach to family support, normalising participation (19, 21). In line with existing literature (41, 42), our findings identified logistical barriers, such as transport, lack of childcare support and difficulty in involving fathers, was limiting attendance and creating challenges in parent recruitment. The studies provided limited insight into the initial sense-making (coherence) among parents, e.g., understanding of the programme's purpose and how it could be relevant to them. This absence of detailed reporting on participants' understanding aligns with Pinto et al., who note that implementation outcomes mostly involve fidelity and acceptability, making it difficult to ascertain why programmes succeed in some settings but not in others (22). Our findings are also consistent with those of Jürgensen et al., who observed that facilitators frequently struggle with recruitment, retention, and parents' perceived relevance of programme content (37). To facilitate engagement, flexible delivery methods and clear communication of the benefits of attending the intervention could be highlighted.

Universal programmes, by design, must engage a more diverse parent population and often operate with limited dedicated resources, which can constrain fidelity and adaptation. Evidence demonstrates that certain subpopulations—including ethnic minorities and parents with low literacy levels—may require culturally and linguistically tailored adaptations to fully benefit from these interventions (43). In our analysis, some health professionals modified their language based on their perception of the resourcefulness of the mother, and stakeholders reported difficulty in engaging, for example, fathers. Jürgensen et al. similarly note that facilitators often adapt content to improve cultural fit and accessibility, although such adaptations can unintentionally dilute core components if not guided by a clear logic model (37). Pinto et al. further emphasise that insufficient documentation of adaptations makes it difficult to assess their impact on programme outcomes (22). The same flexibility that lets universal programmes fit local contexts can, if ungoverned by a clear logic model, dilute the core components responsible for effect. Sustaining them therefore requires distinguishing adaptable surface features from non-negotiable core elements. This reinforces the need for flexible delivery methods combined with structured implementation support, enabling stakeholders to accommodate individual circumstances while maintaining programme coherence. Other systematic reviews using NPT-informed analysis of implementation facilitators and barriers have shown similar findings; with facilitators such as practitioner buy-in and adequate professional skills and barriers such as lack of support and competing workloads (44, 45).

Taken together, our findings contribute to a growing body of evidence suggesting that the sustainability of universal parenting programmes depends on a combination of organisational alignment, facilitator competence, and parent-centred communication strategies. While universal programmes can offer substantial population-level benefits, they require consistent managerial and policy support to be embedded effectively in routine practice. Sustainably delivered universal programmes matter precisely because of their role in early intervention to improve children's mental health.

### Strengths and limitations

We applied a robust, theory-informed synthesis using NPT and systematically assessed reporting transparency with COREQ. The average COREQ score were 15.5 with a median of 17, which is about equal to what is found in other qualitative systematic reviews (46). The application of NPT enabled a theory-informed synthesis, however, categorizing specific findings was at times challenging, as some spanned multiple constructs. Similar issues of overlap in NPT analysis have been noted previously (47). Inclusion of studies across multiple countries and settings strengthened the transferability of findings. However, heterogeneity in intervention formats and the limited representation of parent perspectives in several studies reduce generalisability. Furthermore, the small number of studies highlights the need for continued research on implementation and long-term sustainability of universal programmes as most studies targeted high-risk populations or studied outcomes quantitatively. As the aim (to synthesise experiential and process accounts of how universal programmes become embedded in routine practice) was qualitative by nature, eligibility was restricted to qualitative data; we acknowledge that implementation can also be studied quantitatively, which may be a limitation of this review.

### Implications for research and practice

To enhance sustainability, universal parenting interventions should be co-designed with local stakeholders, integrated with existing health services, and supported by ongoing training and managerial commitment. Routine collection of parent feedback and structured monitoring of adaptations over time could strengthen programme fit and longevity. Future research should focus on:

1. Mechanisms that foster parent engagement across diverse populations,
2. Managerial action—protected time, role clarity and recognition of workload—to support both initial commitment and sustained delivery.
3. Preparing and supporting the workforce to deliver universal programmes within complex, multi-agency service settings
4. Embedding feasible, routine evaluation mechanisms in real-world practice.

## Conclusion

Universal parenting programmes hold promise as scalable public health strategies to strengthen early parent-child relationships and promote family well-being. However, their long-term integration in existing health care settings depends on careful implementation that prioritizes stakeholder engagement, contextual adaptation, and sustained organizational support. By embedding mechanisms for feedback, co-design, and continuous training, these interventions can move beyond pilot phases and deliver meaningful benefits at the population level. Future studies should address persistent engagement barriers and explore strategies for scaling universal interventions while maintaining equity.

## Data Availability

The datasets presented in this study can be found in online repositories. The names of the repository/repositories and accession number(s) can be found in the article/supplementary material.
