# Supplementary material for: Facilitators and barriers to the sustainability of universally accessible parental training interventions: a systematic qualitative review using normalization process theory
Source: Front Pediatr. 2026 Aug 27;14:1802287. doi: 10.3389/fped.2026.1802287 (PMC13558201; doi:10.3389/fped.2026.1802287)
Supplement: Supplementary file 1 [file Table1.docx]

**Additional file 1: Search string for each of the 10 databases.**

| PubMed | ((("Parents"[Mesh]) OR ("Parenting"[Mesh]) OR ("Parent*"[TIAB]) OR ("Primary caregiver"[TIAB]) OR ("Father*"[TIAB]) OR ("Paternal"[TIAB]) OR ("Mother*"[TIAB]) OR ("Maternal"[TIAB])) AND (("Universal access*"[TIAB]) OR ("Public service*"[TIAB]) OR ("Public access*"[TIAB]) OR ("Universal service*"[TIAB]) OR ("Self-refer*"[TIAB]) OR ("Self refer*"[TIAB]) OR ("Primary Care*"[TIAB]) OR ("General Practi*"[TIAB]) OR ("Health Visit*"[TIAB]) OR ("Community Health"[TIAB]) OR (Pediatr*[TIAB]) OR ("Access to Primary Care"[Mesh]) OR ("Primary Care Nursing"[Mesh]) OR ("Primary Health Care"[TIAB]) OR ("Primary Health Care"[Mesh]) OR ("General Practice"[Mesh]) OR ("General Practitioners"[Mesh]) OR ("Pediatric Nursing"[Mesh]) OR ("Pediatrics"[Mesh]) OR ("Nurses, Community Health"[Mesh]))) AND ((("Interact*"[TIAB]) OR ("attach*"[TIAB]) OR ("Psychological Well-Being"[Mesh]) OR ("Psycholog*"[TIAB]) OR ("Sensitiv*"[TIAB]) OR ("Resilien*"[TIAB]) OR ("Self efficacy"[TIAB]) OR ("Self-efficacy"[TIAB]) OR ("Mentali*"[TIAB]) OR ("Well-being"[TIAB]) OR ("Wellbeing"[TIAB]) OR ("Psychological Phenomena"[Mesh]) OR ("Parent Child Relations"[Mesh]) OR (Psychoeduca*[TIAB]) OR ("Psycho-educa*"[TIAB]) OR ("Relation*"[TIAB]))) AND (2004/1/1:2025/7/30[pdat]) AND ((english[Filter]) AND (newborn[Filter] OR infant[Filter] OR preschoolchild[Filter]))  Results:9614 |
| --- | --- |
| PsycInfo | (DE "Parents" OR TI “Parent*” OR AB “Parent*” OR TI “Primary Caregiver” OR AB “Primary Caregiver” OR TI “Father*” OR AB “Father*” OR TI “Paternal” OR AB “Paternal” OR TI “Mother*” OR AB “Mother*” OR TI “Maternal” OR AB “Maternal” ) AND (TI “Public access*” OR AB “Public access*” OR TI “Universal access*” OR AB “Universal access*” OR TI “Public service*” OR AB “Public service*” OR TI “Universal service*” OR AB “Universal service*” OR TI “Self-refer*” OR AB “Self-refer*” OR TI “Self refer*” OR AB “Self refer*” OR DE “Community Welfare Services” OR DE “Self-referral” OR DE “Community Services” OR DE “Public Health Services” OR DE "Primary Health Care" OR TI “primary health care” OR AB “primary health care” OR DE "General Practitioners" OR TI “primary care*” OR AB “primary care*” OR TI “general practi*” OR AB “general practi*” OR TI “health visit*” OR AB “health visit*” OR DE "Community Health" OR DE "Community Mental Health Services" OR DE "Community Welfare Services" OR TI "Community health" OR AB "Community health" OR DE "Pediatricians" OR DE "Pediatrics" OR TI Pediatr* OR AB Pediatr* ) AND (DE "Psychological Development" OR DE “Psychoeducation” OR TI “Psycholog*” OR AB “Psycholog*” OR DE "Sensitivity Training" OR DE “Parent Training” OR DE “Parenting Skills” OR DE “Parent Child Communication” OR DE "Parent Child Relations” OR DE "Self-Help Techniques" OR TI “Interact*” OR AB ”Interact*” OR TI “Attach*” OR AB “Attach*” OR TI “Psychoeduca*” OR AB “Psychoeduca*” OR TI “Psycho-educa*” OR AB “Psycho-educa*” OR TI “Sensitiv*” OR AB “Sensitiv*” OR DE "Resilience (Psychological)" OR TI “resilien*” OR AB “resilien*” OR DE "Self-Efficacy" OR TI “self-efficacy” OR AB “self-efficacy” OR TI “self efficacy” OR AB “self efficacy” OR DE "Mentalization" OR TI “mentali*" OR AB “mentali*" OR TI “Relation*” OR AB “Relation*” OR DE "Well Being" OR TI “Wellbeing” OR AB “Wellbeing” OR TI “Well-being” OR AB “Well-being”)  AND  Filters: English AND (neonatal(birth-1mo) OR (infancy (2-23mo) OR (preschool age (2-5yrs)) AND (01.01.2004-30.07.2025)  Results:2629 |
| SocIndex | (TI “Parent*” OR AB “Parent*” OR TI “Primary Caregiver” OR AB “Primary Caregiver” OR TI “Father*” OR AB “Father*” OR TI “Paternal” OR AB “Paternal” OR TI “Mother*” OR AB “Mother*” OR TI “Maternal” OR AB “Maternal” OR DE "PARENTS") AND (TI “Public access*” OR AB “Public access*” OR TI “Universal access*” OR AB “Universal access*” OR TI “Public service*” OR AB “Public service*” OR TI “Universal service*” OR AB “Universal service*” OR TI “Self-refer*” OR AB “Self-refer*” OR TI “Self refer*” OR AB “Self refer*” OR TI “primary health care” OR AB “primary health care” OR TI “primary care*” OR AB “primary care*” OR TI “general practi*” OR AB “general practi*” OR TI “health visit*” OR AB “health visit*” OR TI "Community health" OR AB "Community health" OR TI Pediatr* OR AB Pediatr* OR DE "UNIVERSAL healthcare" OR DE "COMMUNITY health services" OR DE "PRIMARY health care") AND (TI "Psycholog*" OR AB "Psycholog*" OR TI "Interact*" OR AB "Interact*" OR TI "Attach*" OR AB "Attach*" OR TI "Psychoeduca*" OR AB "Psychoeduca*" OR TI "Psycho-educa*" OR AB "Psycho-educa*" OR TI "Relation*" OR AB "Relation*" OR TI "Sensitiv*" OR AB "Sensitiv*" OR TI "resilien*" OR AB "resilien*" OR TI "self-efficacy" OR AB "self-efficacy" OR TI "self efficacy" OR AB "self efficacy" OR TI "mentali*" OR AB "mentali*" OR TI "Wellbeing" OR AB "Wellbeing" OR TI "Well-being" OR AB "Well-being" OR DE "WELL-being" OR DE "SELF-help techniques" OR DE "PARENT-child relationships")  Filters: (01.01.2004-30.07.2025)  Results: 1421 |
| CINAHL | (TI “Parent*” OR AB “Parent*” OR TI “Primary Caregiver” OR AB “Primary Caregiver” OR TI “Father*” OR AB “Father*” OR TI “Paternal” OR AB “Paternal” OR TI “Mother*” OR AB “Mother*” OR TI “Maternal” OR AB “Maternal” OR (MH "Parents")) AND (TI “Public access*” OR AB “Public access*” OR TI “Universal access*” OR AB “Universal access*” OR TI “Public service*” OR AB “Public service*” OR TI “Universal service*” OR AB “Universal service*” OR TI “Self-refer*” OR AB “Self-refer*” OR TI “Self refer*” OR AB “Self refer*” OR TI “primary health care” OR AB “primary health care” OR TI “primary care*” OR AB “primary care*” OR TI “general practi*” OR AB “general practi*” OR TI “health visit*” OR AB “health visit*” OR TI "Community health" OR AB "Community health" OR TI Pediatr* OR AB Pediatr* OR (MH "Access to Primary Care") OR (MH "Universal Health Care") OR (MH "Primary Health Care") OR (MH "Community Health Centers") OR (MH "Community Health Services")) AND (TI "Psycholog*" OR AB "Psycholog*" OR TI "Interact*" OR AB "Interact*" OR TI "Attach*" OR AB "Attach*" OR TI "Psychoeduca*" OR AB "Psychoeduca*" OR TI "Psycho-educa*" OR AB "Psycho-educa*" OR TI "Relation*" OR AB "Relation*" OR TI "Sensitiv*" OR AB "Sensitiv*" OR TI "resilien*" OR AB "resilien*" OR TI "self-efficacy" OR AB "self-efficacy" OR TI "self efficacy" OR AB "self efficacy" OR TI "mentali*" OR AB "mentali*" OR TI "Wellbeing" OR AB "Wellbeing" OR TI "Well-being" OR AB "Well-being" OR (MH "Psychoeducation") OR (MH "Parent-Child Relations") OR (MH "Psychological Well-Being"))  Filters: (01.01.2004-30.07.2025) AND English AND (fetus, conception to birth OR infant, newborn: birth-1month OR infant:1-23months OR child, preschool: 2-5years)  Results:2930 |
| Embase | (Parent*.ti,ab. or Primary Caregiver.ti,ab. or Father*.ti,ab. or Paternal.ti,ab. or mother*.ti,ab. or maternal.ti,ab. or parent/ or caregiver/) AND (pediatrician/ or pediatrics/ or public health/ or health visitor/ or general practice/ or general practitioner/ or primary health care/ or primary medical care/ or public health service/ or health care access/ or mental health care access/ or primary care access/ or (Universal access* or Public service* or Public access* or Universal service* or self-refer* or self refer* or Primary Care* or Primary Health Care or General Practi* or Health Visit* or Community Health or Pediatr*).ti,ab.) AND (psychoeducation/ or psychological resilience/ or self concept/ or mentalization/ or child parent relation/ or wellbeing/ or positive psychology/ or (Interact* or attach* or Psycholog* or Sensitiv* or Resilien* or Self-efficacy or Self efficacy or Mentali* or Well-being or Wellbeing or Psychoeduca* or Psycho-educa* or Relation*).ti,ab.)  Filter: 2004-2025 AND (infant <to one year> or preschool child <1 to 6 years>”) AND English  Results:6366 |
| Medline | (Parent*.ti,ab or Primary Caregiver.ti,ab. or Father*.ti,ab. or Paternal.ti,ab. or mother*.ti,ab. or maternal.ti,ab. or parent/ or caregiver/) AND (pediatrician/ or pediatrics/ or public health/ or health visitor/ or general practice/ or general practitioner/ or primary health care/ or primary medical care/ or public health service/ or health care access/ or mental health care access/ or primary care access/ or (Universal access* or Public service* or Public access* or Universal service* or self-refer* or self refer* or Primary Care* or Primary Health Care or General Practi* or Health Visit* or Community Health or Pediatr*).ti,ab.) AND  (psychoeducation/ or psychological resilience/ or self concept/ or mentalization/ or child parent relation/ or wellbeing/ or positive psychology/ or (Interact* or attach* or Psycholog* or Sensitiv* or Resilien* or Self-efficacy or Self efficacy or Mentali* or Well-being or Wellbeing or Psychoeduca* or Psycho-educa* or Relation*).ti,ab.)  Filter: 2004-2025 AND (all infant (0-23mo) or preschool (2-5years)) AND English  Results:5758 |
| Sociological abstract | (MAINSUBJECT.EXACT("Parents & parenting") OR title(parent*) OR abstract(parent*) OR title(primary caregiver) OR abstract(primary caregiver) OR title(Father*) OR abstract(Father*) OR title(Paternal) OR abstract(Paternal) OR title(mother*) OR abstract(mother*) OR title(Maternal) OR abstract(Maternal)) AND (MAINSUBJECT.EXACT("Pediatrics") OR MAINSUBJECT.EXACT("Primary care") OR MAINSUBJECT.EXACT("Pediatricians") OR MAINSUBJECT.EXACT("Community health services") OR title("Universal access*") OR abstract("Universal access*") OR title("Public service*") OR abstract("Public service*") OR title("Universal service*") OR abstract("Universal service*") OR title("Public access*") OR abstract("Public access*") OR title("Self refer*") OR abstract("Self refer*”) OR title("Self-refer*") OR abstract("Self-refer*") OR title("Primary care*") OR abstract("Primary care*") OR title("Primary health care") OR abstract("Primary health care") OR title("General practi*") OR abstract("General practi*") OR title("Health visit*") OR abstract("Health visit*") OR title("Community Health") OR abstract("Community Health") OR title(Pediatr*) OR abstract(Pediatr*)) AND (title("Relation*") OR abstract("Relation*") OR title("Wellbeing") OR abstract("Wellbeing") OR title("Well-being") OR abstract("Well-being") OR title(Sensitiv*) OR abstract(Sensitiv*) OR title(Resilien*) OR abstract(Resilien*) OR title("Self efficacy") OR abstract("Self efficacy") OR title("Self-efficacy") OR abstract("Self-efficacy") OR title("Mentali*") OR abstract("Mentali*") OR title(Interact*) OR abstract(Interact*) OR title(Attach*) OR abstract(Attach*) OR title("Psychoeduca*") OR abstract("Psychoeduca*") OR title("Psycho-educa*") OR abstract("Psycho-educa*") OR title("Psycholog*") OR abstract("Psycholog*") OR MAINSUBJECT.EXACT("Attachment") OR MAINSUBJECT.EXACT("Resilience") OR MAINSUBJECT.EXACT("Well being") OR MAINSUBJECT.EXACT("Social interaction") OR MAINSUBJECT.EXACT("Self-efficacy") OR MAINSUBJECT.EXACT("Sensitivity training")) AND (MAINSUBJECT.EXACT("Children") OR MAINSUBJECT.EXACT("Infants"))  Filters: (01.01.2004-30.07.2025) AND English  Results:951 |
| ERIC | (title(parent*) OR abstract(parent*) OR title(primary caregiver) OR abstract(primary caregiver) OR title(Father*) OR abstract(Father*) OR title(Paternal) OR abstract(Paternal) OR title(mother*) OR abstract(mother*) OR title(Maternal) OR abstract(Maternal) OR MAINSUBJECT.EXACT("Parents")) AND (title("Universal access*") OR abstract("Universal access*") OR title("Public service*") OR abstract("Public service*") OR title("Universal service*") OR abstract("Universal service*") OR title("Public access*") OR abstract("Public access*") OR title("Self refer*") OR abstract("Self refer*") OR title("Self-refer*") OR abstract("Self-refer*") OR title("Primary care*") OR abstract("Primary care*") OR title("Primary health care") OR abstract("Primary health care") OR title("general practi* ") OR abstract("general practi*") OR title("health visit*") OR abstract("health visit*") OR title("Community Health") OR abstract("Community Health") OR title(Pediatr*) OR abstract(Pediatr* ) OR MAINSUBJECT.EXACT("Primary Health Care") OR MAINSUBJECT.EXACT("Public Service") OR MAINSUBJECT.EXACT("Pediatrics") OR MAINSUBJECT.EXACT("Community Health Services") OR MAINSUBJECT.EXACT("Family Practice (Medicine)")) AND (title("Relation*") OR abstract("Relation*") OR title("Wellbeing") OR abstract("Wellbeing") OR title("Well-being") OR abstract("Well-being") OR title(Sensitiv*) OR abstract(Sensitiv*) OR title(Resilien*) OR abstract(Resilien*) OR title("Self-efficacy") OR abstract("Self-efficacy") OR title("Self efficacy") OR abstract("Self efficacy") OR title("Mentali*") OR abstract("Mentali*") OR title(Interact*) OR abstract(Interact*) OR title(Attach*) OR abstract(Attach*) OR title("Psychoeduca*") OR abstract("Psychoeduca*") OR title("Psycho-educa*") OR abstract("Psycho-educa*") OR title("Psycholog*") OR abstract("Psycholog*") OR MAINSUBJECT.EXACT("Self Efficacy") OR MAINSUBJECT.EXACT("Sensitivity Training") OR MAINSUBJECT.EXACT("Parent Child Relationship") OR MAINSUBJECT.EXACT("Parenting Skills") OR MAINSUBJECT.EXACT("Well Being") OR MAINSUBJECT.EXACT("Resilience (Psychology)") OR MAINSUBJECT.EXACT("Interaction")) AND (MAINSUBJECT.EXACT("Children"))  Filters: (01.01.2004-30.07.2025) AND English  Results:186 |
| Scopus*  Two search strings have been included | ((TITLE-ABS-KEY({Parent}) OR TITLE-ABS-KEY({Mother}) OR TITLE-ABS-KEY({Father}) OR TITLE-ABS-KEY({Maternal}) OR TITLE-ABS-KEY({Paternal}) OR TITLE-ABS-KEY({Primary Caregiver}))) AND ((TITLE-ABS-KEY({Interact*}) OR TITLE-ABS-KEY({Attach*}) OR TITLE-ABS-KEY({Psycholog*}) OR TITLE-ABS-KEY({Sensitiv*}) OR TITLE-ABS-KEY({Resilien*}) OR TITLE-ABS-KEY({Self-efficacy}) OR TITLE-ABS-KEY({Mentali*}) OR TITLE-ABS-KEY({Wellbeing}) OR TITLE-ABS-KEY({Well-being}))) AND ((TITLE-ABS-KEY({Universal access}) OR TITLE-ABS-KEY({Public service}) OR TITLE-ABS-KEY({Public access}) OR TITLE-ABS-KEY({Self-refer*}) OR TITLE-ABS-KEY({Primary Care}) OR TITLE-ABS-KEY({Primary Health Care}) OR TITLE-ABS-KEY({General practi*}) OR TITLE-ABS-KEY({Health visit*}) OR TITLE-ABS-KEY({Community Health}) OR TITLE-ABS-KEY({Pediatr*}))) AND PUBYEAR > 2003 AND PUBYEAR < 2026  Results: 967  ____  (TITLE-ABS-KEY(implement* ) OR TITLE-ABS-KEY(sustain*))) AND (((TITLE-ABS-KEY("Parent*") OR TITLE-ABS-KEY("Mother*") OR TITLE-ABS-KEY("Father*") OR TITLE-ABS-KEY("Maternal") OR TITLE-ABS-KEY("Paternal") OR TITLE-ABS-KEY("Primary Caregiver")) AND (TITLE-ABS-KEY("Universal access*") OR TITLE-ABS-KEY("Public service*") OR TITLE-ABS-KEY("Public access*") OR TITLE-ABS-KEY ("Universal service*" ) OR TITLE-ABS-KEY ( "Self-refer*") OR TITLE-ABS-KEY("Self refer*") OR TITLE-ABS-KEY("Primary Care*") OR TITLE-ABS-KEY("Primary Health Care") OR TITLE-ABS-KEY("General practi*") OR TITLE-ABS-KEY("Health visit*") OR TITLE-ABS-KEY("Community Health") OR TITLE-ABS-KEY("Pediatr*")) AND (TITLE-ABS-KEY("Interact*") OR TITLE-ABS-KEY("Attach*") OR TITLE-ABS-KEY("Psycholog*") OR TITLE-ABS-KEY("Sensitiv*") OR TITLE-ABS-KEY("Resilien*”) OR TITLE-ABS-KEY("Self efficacy") OR TITLE-ABS-KEY("Self-efficacy") OR TITLE-ABS-KEY("Mentali*") OR TITLE-ABS-KEY("psychoeduca*" ) OR TITLE-ABS-KEY("Psycho-educa*" ) OR TITLE-ABS-KEY("Relation*") OR TITLE-ABS-KEY("Wellbeing") OR TITLE-ABS-KEY("Well-being"))) AND (LIMIT-TO (EXACTKEYWORD , "Preschool Child" ) OR LIMIT-TO(EXACTKEYWORD , "Child, Preschool") OR LIMIT-TO (EXACTKEYWORD , "Infant") OR LIMIT-TO (EXACTKEYWORD, "Infant, Newborn") OR LIMIT-TO (EXACTKEYWORD , "Newborn")) AND PUBYEAR > 2003 AND PUBYEAR < 2026  Results: 2189 |
| Web of Science | (AB=(Parent*) OR TI=(Parent*) OR TI=("Primary caregiver") OR AB=("Primary caregiver") OR AB=(Father*) OR TI=(Father*) OR TI=(Mother*) OR AB=(Mother*) OR AB=(Paternal) OR TI=(Paternal) OR TI=(Maternal) OR AB=(Maternal)) AND (TI=("Universal Access*") OR AB=("Universal access*") OR TI=("Universal Service*") OR AB=("Universal Service*") OR AB=("Public Service*") OR TI=("Public Service*") OR TI=("Public Access*") OR AB=("Public Access*") OR AB=("Self-refer*") OR TI=("Self-refer*") OR AB=("Self refer*") OR TI=("Self refer*") OR AB=("Primary Care*") OR TI=("Primary Care*") OR TI=("Primary Health Care") OR AB=("Primary Health Care") OR AB=("General Practi*") OR TI=("General Practi*") OR TI=("Health Visit*") OR AB=("Health Visit*") OR AB=("Community Health") OR TI=("Community Health") OR TI=(Pediatr*) OR AB=(Pediatr*)) AND (TI=(Interact*) OR AB=(Interact*) OR AB=(Attach*) OR TI=(Attach*) OR TI=(Psycholog*) OR AB=(Psycholog*) OR AB=(Sensitiv*) OR TI=(Sensitiv*) OR TI=(Resilien*) OR AB=(Resilien*) OR AB=("Self efficacy") OR TI=("Self efficacy") OR AB=("Self-efficacy") OR TI=("Self-efficacy") OR TI=(Mentali*) OR AB=(Mentali*) OR AB=(Wellbeing*) OR TI=(Wellbeing*) OR TI=(Well-being*) OR AB=(Well-being*) OR AB=(“Parent Child Relation*”) OR TI=(“Parent Child Relation*”) OR TI=(Relation*) OR AB=(Relation*) OR TI=(Psychoeduca*) OR AB=(Psychoeduca*) OR TI=(Psycho-educa*) OR AB=(Psycho-educa*))  Refine results: Infant or preschool AND 2004-2025  Results:2357 |
